# First-Principles Study on the Mechanical Properties and Electronic Structure of V Doped WCoB and W_2_CoB_2_ Ternary Borides

**DOI:** 10.3390/ma12060967

**Published:** 2019-03-22

**Authors:** Tong Zhang, Haiqing Yin, Cong Zhang, Ruijie Zhang, Xue Jiang, Qingjun Zheng, Xuanhui Qu

**Affiliations:** 1 Institute for Advanced Materials and Technology, University of Science and Technology Beijing, Beijing 100083, China; tobyzhang1992@163.com (T.Z.); quxh@ustb.edu.cn (X.Q.); 2 Collaborative Innovation Center of Steel Technology, University of Science and Technology Beijing, Beijing 100083, China; zhangcong@ustb.edu.cn (C.Z.); zrj@ustb.edu.cn (R.Z.); jiangxue@ustb.edu.cn (X.J.); 3 Beijing Laboratory of Metallic Materials and Processing for Modern Transportation, University of Science and Technology Beijing, Beijing 100083, China; 4 Beijing Key Laboratory of Materials Genome Engineering, University of Science and Technology Beijing, Beijing 100083, China; 5 Kennametal Incorporated Company, 1600 Technology Way, Latrobe, PA 15650, USA; jack.zheng@kennametal.com; 6 Beijing Advanced Innovation Center for Materials Genome Engineering, University of Science and Technology Beijing, Beijing 100083, China

**Keywords:** first-principles calculations, V doping, WCoB, W_2_CoB_2_, electronic structure

## Abstract

For the purpose of exploring new hard materials and doping methods, the structural, mechanical and electronic properties of WCoB and W_2_CoB_2_ ternary boride were investigated with 0, 8.33, 16.67, 25 and 33.33 at.% V doping content and W_2_CoB_2_ with 0, 5, 10, 15 and 20 at.% V doping content by first-principle calculations. The cohesive energy, impurity formation energy and formation energy indicate the structural stability of V doped WCoB and W_2_CoB_2_. The elastic constants and mechanical properties imply that V doping leads to the decrement of shear modulus and the increment of ductility. Two different kinds of hardness models verify that V doping contributes to the decrement of hardness, which is closely related to shear modulus. The electronic structure is analyzed by DOS (density of states), PDOS (partial density of states) and charge density difference, which indicate the formation of weaker B–V covalent bonds, W–V and W–W metallic bonds lead to the decrement of mechanical properties. Compared with previous studies of Cr, Mn doped WCoB and W_2_CoB_2_, V doping leads to worse mechanical properties and hardness, indicating V may not be a suitable choice of doping transition elements.

## 1. Introduction

Ternary borides, including Mo_2_FeB_2_, Mo_2_NiB_2_, WCoB, were fabricated by reaction bronizing sintering, which solved the poor sintering property of binary borides [1]. Ternary borides are suitable substitutes for WC-Co cermet owing to their high melting point, high hardness, high corrosion resistance and high electrical conductivity [2]. For their great potential as a wear-resistant material, ternary borides have attracted scientists’ attention in recent years [3,4,5,6].

Due to its outstanding mechanical properties, WCoB ternary boride has been studied for years [7,8,9]. Current studies of ternary borides are focused on the transition elements of doping to improve mechanical properties, such as V, Cr, Mn, Nb, etc. [10,11,12,13,14]. V is an important choice of transition element doping and is widely used to improve the mechanical properties of materials [15,16,17,18,19,20,21,22]. Hu [23] and Yu [24] found 2 wt.% V doping is able to improve the hardness and transverse rupture strength (TRS) of Mo_2_FeB_2_ up to 87 HRA and 1800 MPa, respectively. Shiota [25], Yamasaki [26] and Takagi [27] found V doping can improve the wettability of the Mo_2_NiB_2_ hard phase, hardness and TRS up to 90.8 HRA (Rockwell hardness A) and 3200 MPa. This enhancement is owed to grain refinement, decrease of porosity and increase of phase uniformity. Yamasaki [28] also found 12.5 wt.% V doping leads to Mo_2_NiB_2_ phase transformation from orthorhombic phase to tetragonal phase and TRS up to 2500 MPa with the smallest grain size.

However, experimental studies of V doped WCoB ternary boride are rare. The first-principle calculation has been widely used to explore mechanical properties and electronic structure, which can provide more information on experiments. Yang [29] and Sun [30] used first-principle calculation to find Mn doping improves the mechanical properties of Mo_2_FeB_2_ ternary boride and verified the results by experiment with the highest hardness and TRS up to 89.4 HRA and 1290 MPa, respectively. Wang [31] and Lin [32] studied the effects of different contents of Cr, Ni, Mn doped Mo_2_FeB_2_ ternary boride. They found Ni doping leads to the increment of ductility and elastic modulus and Cr doping contributes to the increment of bulk modulus. Li [33] studied the different contents of V doped Mo_2_NiB_2_ by first-principle calculation and found that Mo_1.625_Ni_0.625_V_0.75_B_2_ has the lowest energy.

Because there are no reports on V doped WCoB, we used first-principle calculation to explore the effect of V doping on benefitting the selection of a suitable doping element. WCoB and W_2_CoB_2_ are regular hard phase in WCoB ternary boride hard alloy [34], so two cases are considered: the content of V doping are 0, 8.33, 16.67, 25 and 33.33 at.% separately in WCoB unit cell and 0, 5, 10, 15 and 20 at.% separately in W_2_CoB_2_ super cell.

It is worth pointing out that the effects of different contents of Cr, Mn doped WCoB on mechanical properties and electronic structure have been studied by first-principle calculation in our previous work [35,36]. We found that B–Cr, B–Mn bonds play an important role in the crystal. Nevertheless, the previous studies of Cr, Mn doping WCoB is calculated by unit cell, which cannot provide sufficient details on the variation of W_2_CoB_2_ in different doping contents. So, the structure, lattice parameters, population, density of states and charge density difference of WCoB and W_2_CoB_2_ are studied in detail to explore their mechanical properties and hardness in different V doped structures. At the same time, the mechanical properties and hardness of Cr, Mn, V doped WCoB and W_2_CoB_2_ are also discussed.

## 2. Crystal Structure and Calculation Method

All first-principle calculations in this paper are conducted based on density functional theory (DFT) [37,38] with Cambridge Serial Total Energy Package (CASTEP) code [39]. The exchange and correlation terms are described by generalized gradient approximation (GGA) in the Perdew-Burke-Ernzerhof (PBE) form. Different Monkhorst-Pack nets have been evaluated to ensure the lowest energy of different models. The interaction of the valence electrons and ionic cores are calculated with Vanderbilt ultrasoft pseudopotential. The valence states considered here are chosen as W (5s^2^5p^6^5d^4^6s^2^), Co (3d^7^4s^2^), B (2s^2^2p^1^) and V (3s^2^4p^6^3d^3^4s^2^). The integration over the Brillouin zone was conducted with Monkhorst and Pack k-point mesh integrations.

**Figure 1:**
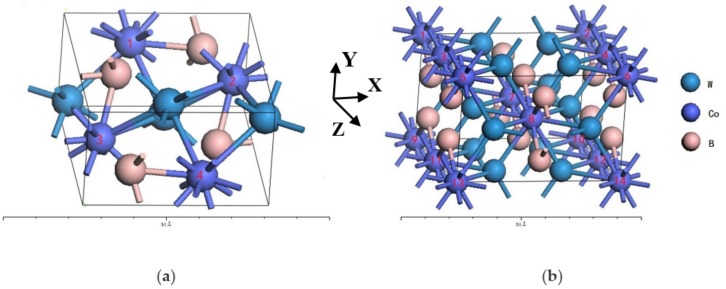
Orthorhombic structure of V doping (**a**) WCoB (W_4_Co_4−*x*_V*_x_*B_4_, *x* = 0, 1, 2, 3, 4) and (**b**) W_2_CoB_2_ (W_8_Co_4−*x*_V*_x_*B_4_, *x* = 0, 1, 2, 3, 4).

The space group of orthorhombic WCoB (ICSD collection code: 613390) [40] is No.62 Pnma. The Wyckoff positions of W, Co and B are 4c (0.021, 0.25, 0.18), 4c (0.142, 0.25, 0.561) and 4c (0.765, 0.25, 0.623). The lattice parameters are a = 5.745 Å, b = 3.203 Å and c = 6.652 Å. The WCoB unit cell that contains 4 W atoms, 4 Co atoms and 4 B atoms with periodic boundary conductions is used, as shown in Figure 1. The effect of V doping content for WCoB is calculated by using different numbers of V atoms to replace Co site positions. The chemical formulae can be expressed by: (a) W_4_Co_4_B_4_, (b) W_4_Co_3_VB_4_ (position: 1), (c) W_4_Co_2_V_2_B_4_-sy (position: 1, 4); W_4_Co_2_V_2_B_4_-unsy (position: 1, 2), (d) W_4_CoV_3_B_4_ (position: 1, 2, 4) and (e) W_4_V_4_B_4_ (all position of Co atoms) by replacing 0, 1, 2, 3 and 4 Co atoms in WCoB unit-cell.

The space group of orthorhombic W_2_CoB_2_ (ICSD collection code: 16776) [41] is No.71 Immm. The Wyckoff positions of W, Co and B are 4f (0.205, 0.5, 0), 2a (0, 0, 0) and 4h (0, 0.3, 0.5). The lattice parameters are a = 7.075 Å, b = 4.564 Å and c = 3.177 Å. To investigate the influence of different doping content, we constructed supercell 1 × 1 × 2 containing 8 W atoms, 4 Co atoms and 8 B atoms with periodic boundary conductions, as shown in Figure 1. The effect of V doping content for W_2_CoB_2_ is calculated by using different numbers of V atoms to replace Co site positions. The chemical formulae can be expressed by (f) W_8_Co_4_B_8_, (g) W_8_Co_3_VB_8_ (position: 8), (h) W_8_Co_2_V_2_B_8_ (position: 7, 8), (i) W_8_CoV_3_B_8_ (position: 3, 4, 7, 8, 11, 12) and (j) W_8_V_4_B_8_ (all positon of Co atoms) by replacing 0, 1, 2, 3 and 4 Co atoms in W_2_CoB_2_ super cell.

The cutoff energy of a plane-wave set was set as 600 eV and 400 eV to calculate electron wave function, and the k-point grid was set as 5 × 10 × 5 and 3 × 5 × 4 for WCoB and W_2_CoB_2_, respectively. The Broyden-Flecher-Goldfarb-Shanno (BFGS) geometry optimization task was applied to obtain a fully relaxed atomic position and a stable structure with minimum total energy [42] during geometry optimization. The convergence conditions were set as the maximum stress below 0.01 GPa, the maximum force on the atom below 0.01 eV/Å, the self-consistent convergence of the total energy below 5 × 10^−6^ eV/atom and the maximum displacement between cycles below 5 × 10^−4^ Å.

## 3. Results and Discussion

### 3.1. Structural Stability

In order to explore the effects of V doping on the mechanical properties and electronic structure of WCoB and W_2_CoB_2_, we first examine the stability of a lattice with different V doping contents. To reveal the stability, the cohesive energy (E_coh_) has been calculated, which is defined as follows:

(1) $$E_{\mathrm{coh}}\;=\;\left(E_{\mathrm{total}}-{\mathrm{aE}}_{w}-{\mathrm{bE}}_{\mathrm{Co}}-{\mathrm{cE}}_{B}-{\mathrm{dE}}_{V}\right)/\left(a+b+c+d\right)$$

where E_coh_ is the cohesive energy of V doped WCoB and W_2_CoB_2_; E_total_ is the total energy of V doped WCoB/W_2_CoB_2_; E_W_, E_Co_, E_B_ and E_V_ are the energy of an isolated atoms; a, b, c and d are the numbers of W, Co, B, V atoms in the WCoB or W_2_CoB_2_ lattice, respectively.

The impurity formation energy (E_f_) can be used to evaluate the change of stability before and after doping, which is given as follows:

(2) $$E_{f}\;={\;E}_{\mathrm{total}}-E_{\mathrm{WCoB}/W_{2}{\mathrm{CoB}}_{2}}+{\mathrm{xE}}_{\mathrm{Co}}-{\mathrm{xE}}_{V}$$

where E_f_ is impurity formation energy (E_f_); E_WCoB/W__2CoB__2_ is the total energy of undoped WCoB and W_2_CoB_2_; x is the number of V replacing Co atoms in different V doped WCoB and W_2_CoB_2_.

The formation enthalpy is the criterion to estimate the difficulty of formation of V doped WCoB/W_2_CoB_2_ from a simple substance, which is shown as follows:

(3) $$\Delta H\;={\;E}_{\mathrm{coh}}\left(\mathrm{cell}\right)-{\mathrm{aE}}_{\mathrm{coh}}\left(W\right)-{\mathrm{bE}}_{\mathrm{coh}}\left(\mathrm{Co}\right)-{\mathrm{cE}}_{\mathrm{coh}}\left(B\right)-{\mathrm{dE}}_{\mathrm{coh}}\left(V\right)$$

where ΔH is the formation enthalpy; E_coh_(cell) is the cohesive energy of V doped WCoB/W_2_CoB_2_; E_coh_(W), E_coh_(Co), E_coh_(B) and E_coh_(V) are the cohesive energy of W, Co, B and V in simple substance W (IM3M), Co (P64MMC), B (P4N2) and V (IM3M).

The calculated values of lattice parameters, unit-cell volumes, cohesive energy, impurity formation energy and formation enthalpy of V doped WCoB and W_2_CoB_2_ are listed in Table 1, with the experimental data for comparison. The crystal structure of V doped WCoB and W_2_CoB_2_ was studied by replacing Co atoms with different numbers of V atoms, and the calculated results are consistent with the experimental data, which proves the stability of the present calculations.

The lattice parameters and the volume of the unit cell show that V doping leads to a change of lattice parameters and an increase in volume. The atom radius of V (r = 171 pm) is larger than the atom radius of Co (r = 152 pm). The difference of the radius is less than 15%, which is in accordance with the criterion of doping. The volume changes of V doped WCoB are (b) 2.69%, (c-sy) 5.09%, (c-unsy) 5.29%, (d) 7.75% and (e) 9.88%, respectively. The volume changes of V doped W_2_CoB_2_ are (g) 1.75%, (h) 3.19%, (i) 4.94% and (j) 6.42%, respectively and the similar variation trend can be found in the change of the lattice parameters.

Equation (1) requires negative values of E_coh_, which refers to a thermodynamically stable structure. With an increase of V doping content, the cohesive energy of WCoB decreases slightly except W_4_Co_3_VB_4_, which indicates that V doping leads to the increment of stability. For V doped W_2_CoB_2_, a higher V doping content contributes to the increment of cohesive energy except W_8_V_4_B_8_, but all the cohesive energy is less than zero, which indicates that V doping decreases stability.

Impurity formation energy is calculated for V doped WCoB and W_2_CoB_2_, which can examine the stability of the doped structure, comparing undoped WCoB and W_2_CoB_2_. The positive number of impurity formation energy means that V doping leads to the decrement of the stability, and the negative number means that V doping leads to the increment of the stability. It is obvious that the V doping leads to a thermodynamically stable state except W_4_Co_3_VB_4_, but V doped W_2_CoB_2_ are more unstable than W_2_CoB_2_ except W_8_V_4_B_8_, and these results are consistent with the analysis of cohesive energy.

Formation enthalpy can be used as the standard of difficulty in a simple substance transforming into compounds. All the values of formation enthalpy are negative, and the increment of formation enthalpy indicates that V doping leads to the increasing of difficulty of transformation from simple substance to WCoB structure. However, when the V doping content reaches the maximum value, the formation enthalpy decreases slightly, which indicates a new stable structure W_4_V_4_B_4_ and W_8_V_4_B_8_ is more easily to form. However, the first-principle calculation is calculated in 0 K and 0 GPa, which needs further study on the thermodynamic formation process.

Overall, it can be concluded that all V doped structures retain a stable state. The unsymmetrical structure W_4_Co_2_V_2_B_4_ is more unstable than the symmetrical structure W_4_Co_2_V_2_B_4_, which will not be considered in the next section.

### 3.2. Mechanical Properties

**Figure 2:**
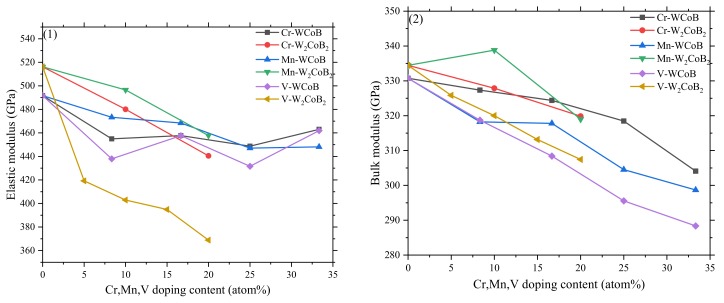

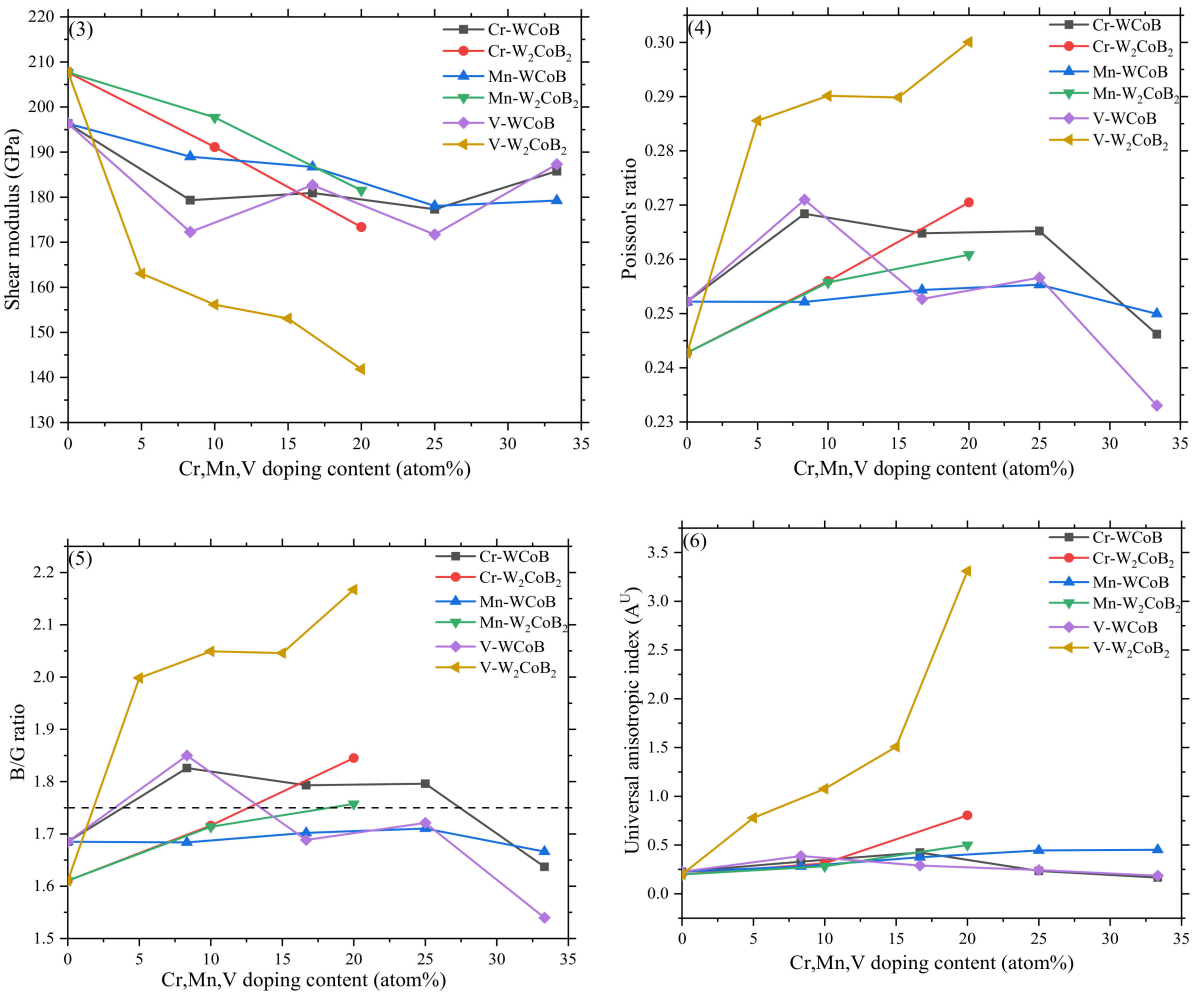
Calculated values of (**1**) Elastic modulus E (GPa); (**2**) Bulk modulus B (GPa); (**3**) Shear modulus G (GPa); (**4**) Poisson’s ratio  γ; (**5**) B/G ratio; and (**6**) universal anisotropic index (A^U^) of W_4_Co_4−*x*_(Cr, Mn, V)*_x_*B_4_ and W_8_Co_4−*x*_(Cr, Mn, V)*_x_*B_8_ (*x* = 0, 1, 2, 3, 4).

The mechanical properties of different contents of V doped WCoB and W_2_CoB_2_, including the elastic constants, bulk modulus, elastic modulus, shear modulus, B/G ratio, Poisson’s ratio, and anisotropic index, are shown in Table 2 and Table 3 and Figure 2. The traditional mechanical stability conditions should be taken into consideration before the analysis of mechanical properties. For orthorhombic crystals WCoB and W_2_CoB_2_, there are nine independent elastic stiffness constants, and the standards can be expressed as follows:

(4) $$c_{\mathrm{ii}}>0,\left[c_{11}+c_{22}+c_{33}+2\left(c_{12}+c_{13}+c_{23}\right)\right]>0,(c_{11}+c_{22}-2c_{12})>0,\;\left(c_{11}+c_{33}-2c_{13}\right)>0,\left(c_{22}+c_{33}-2c_{23}\right)>0$$

where C_ij_ is the single crystal elastic constant. It is obvious that all structures satisfy the stability standards. The mechanical properties of different structures are characterized by the set of elastic constants. For orthorhombic structures WCoB and W_2_CoB_2_, B_V_, B_R_, G_V_, and G_R_ can be expressed by using the following equations:

(5) $$B_{R}\;=\;1/\left[(S_{11}+S_{22}+S_{33}\right)+2\left(S_{12}+S_{13}+S_{23}\right)]$$

(6) $$B_{V}\;=\;\left(C_{11}+C_{22}+C_{33}\right)/9+2\left(C_{12}+C_{13}+C_{23}\right)/9$$

(7) $$G_{R}\;=\;15/\left[4(S_{11}+S_{22}+S_{33}\right)-4\left(S_{12}+S_{13}+S_{23}\right)+3\left(S_{44}+S_{55}+S_{66}\right)]$$

(8) $$B_{V}\;=\;\left(C_{11}+C_{22}+C_{33})/15-(C_{12}-C_{13}-C_{23}\right)/15+\left(C_{44}+C_{55}+C_{66}\right)/5$$

where S_ij_ is the elastic compliant coefficient, which can be converted from the corresponding C_ij_ matrix equation. Bulk modulus B and shear modulus G are calculated by Voigt-Reuss-Hill approximations [43], which are shown in Equations (9) and (10). According to the structural symmetry, Young’s modulus E and Poisson’s ratio  υ  can also be calculated by Equations (11) and (12):

(9) $$B\;=\;(B_{V}+B_{R})/2$$

(10) $$G\;=\;\left(G_{V}+G_{R}\right)/2$$

(11) $$E\;=\;\left(9\mathrm{BG}\right)/\left(3B+G\right)$$

(12) $$\upsilon \;=\;\left(3B-2G\right)/\left[2\left(3B+G\right)\right]$$

A universal elastic anisotropy index (A^U^) can also be calculated as follows, which are used to account for the shear and bulk contributions.

(13) $$A^{U}\;=\;5G_{V}/G_{R}+{\;B}_{V}/B_{R}-6\geq 0$$

The elastic constants of V doped WCoB and W_2_CoB_2_ are shown in Table 2 and Table 3, which satisfy the Born stability criteria for orthorhombic crystals [44,45]. For orthorhombic WCoB ternary boride, it is obvious that C_11_, C_22_, C_33_ decrease with the increasing of V doping content, indicating that V doping contributes to the decreasing of deformation resistance along a, b, c axes. Almost all the C_11_ are higher than C_33_, which demonstrates that the deformation resistance along the a axis is stronger than that along the c axis, which is attributed to the stronger B–Co bonds. For orthorhombic W_2_CoB_2_ ternary boride, C_22_ is larger than C_11_ and C_33_, implying that b axis has the largest stiffness. Although all elastic constants decrease with the increase of V doping content, C_66_ has the biggest decreasing amplitude among all elastic constants. The phenomenon shows that the stiffness obviously decreases along the XZ direction, which is attributed to the bigger atom radius of V and the weaker bonds of W–V metallic bonds.

According to the elastic constants, the calculated values of shear modulus G, bulk modulus B, Young’s modulus E, Poisson’s ratio γ, B/G ratio and universal anisotropic index (A^U^) of Cr, Mn, V WCoB and W_2_CoB_2_ are shown in Figure 2. It is an obvious bulk modulus of WCoB and W_2_CoB_2_ decreases slightly with the increase of the V doping contents, implying that V doping leads to the increase of stress concentration and the decrease of deformation resistance. The calculated values of shear modulus and elastic modulus show a similar trend with variations of the V doping content. For orthorhombic WCoB, V doping leads to the decrease of shear modulus and elastic modulus, and symmetrical structures have higher values. V doping leads to the sharp decrement of shear modulus and elastic modulus for W_2_CoB_2_ ternary boride.

The brittleness can be evaluated by Poisson’s ratio [46]. Poisson’s ratio fluctuates around 0.25 and decreases slightly for WCoB ternary boride, which means V doping contributes to a decrease in ductility. V doping contributes to the obvious increment of Poisson’s ratio of W_2_CoB_2_, implying that V doping leads to an increase of ductility. Generally speaking, the 1.75 ratio of B/G is the criterion with which evaluate the ductility of materials [47]. If B/G is less than 1.75, a material can be determined a brittle material, and with values higher than 1.75 it can be considered a ductile material. So, the B/G ratio is in consistent with the analysis of Poisson’s ratio. V doping leads to the slight decrement of ductility of WCoB ternary boride and the obvious increment in W_2_CoB_2_ ternary boride. The results indicate that V doping leads to an increase of metallicity. A universal elastic anisotropy index (A^U^) is the criterion with which to evaluate the anisotropy. Because the departure of A^U^ from 0 means an increase of anisotropy, it is obvious that V doping leads to the increment of anisotropy for W_2_CoB_2_ ternary boride. However, the variation of A^U^ of WCoB is minor, which indicates that the effect of V doping is more obvious on the supercell of W_2_CoB_2_.

Based on our previous work [35,36], the different contents of Cr, Mn doped WCoB and W_2_CoB_2_ have been studied by first-principle calculation. Compared with the previous data, it is obvious that the low content of Cr, Mn, V doping shows relatively higher mechanical properties, especially on the bulk modulus and the B/G ratio. Ductility is one of the most important properties, and the low content of Cr, V doped WCoB shows the best ductility except for V doped W_2_CoB_2_. Although V doped W_2_CoB_2_ shows the best ductility, the decrement of mechanical properties limits its application.

### 3.3. Population Analysis and Hardness

The chemistry bond plays a crucial role in the mechanical properties, especially on the hardness of the hard phase. The bonding strength among atoms shows information about hardness and is determined by the overlap population [48]. The covalent bonds have a larger overlap population than ionic bonds, which are bigger than zero. Anti-bonding exists when the overlap population is less than zero. The properties of chemical bonds can be analyzed by the average overlap population, which is provided by Zhou [49], and the equation is as follows:

(14) $$n_{\mathrm{AB}}\;=\;\frac{\sum_{i}n_{i}^{\mathrm{AB}}N_{i}}{\sum_{i}N_{i}}$$

where N_i_ is the total number of A-B bonds and $n_{i}^{\mathrm{AB}}$ is the bond population of A-B bond of the i type.

The calculated results of the average overlap population of different bonds in WCoB and W_2_CoB_2_ ternary boride is listed in Table 4 and Table 5. For WCoB ternary boride, V doping leads to an increase of average overlap population, implying V doping contributes to the increasing of covalent properties for most bonds. Compared with W_2_CoB_2_ ternary boride, there are no existing B–B bonds in the V doped WCoB ternary boride, which is attributed to the long distance between B atoms. The negative average overlap population means the repulsion force among atoms, and all the negative value appears between metal atoms. Similarly, average overlap population increases with the increase of V doping content in W_2_CoB_2_ ternary boride. It is clear that V doping contributes to the increase of covalent properties for most bonds. However, the population of B–V and Co–V bonds is less than B–Co and Co–Co bonds, so the V doping leads to a decrease of covalent properties for the WCoB and W_2_CoB_2_ structure.

Hardness is one of the most important properties for hard alloy, and different hardness models are employed to study the influence of V doping on WCoB and W_2_CoB_2_ ternary boride. Hardness is a macroscopic property, which is affected by different factors. Some hardness models are based on mechanical properties, and some hardness models are based on population analysis. To explore the hardness of WCoB and W_2_CoB_2_ ternary boride, the Vickers hardness (H_V_) is calculated by three hardness models.

Pugh [50] proposed a simple hardness model in 1954, which can be expressed by H_V_ = 0.151G. Based on previous models, Chen [51] introduces the Pugh’s ratio to evaluate the ductility of materials, which can be expressed as k = G/B. The hardness model can be expressed as follows:

(15) $$H_{v}\;=\;2{\left(k^{2}G\right)}^{0.585}-3$$

where k is the Pugh’s ratio and G is the shear modulus.

As we know, the mechanical properties are the macroscopic properties, and the previous hardness model only shows the statistical relationship between hardness and mechanical properties. Gao [52] established a new hardness model, which is based on population analysis. Gao proposes that the resistance is proportional to the homo-polar energy gap and the bond strength is determined by overlap populations, which can be evaluated by first-principle calculations. The hardness of bonds can be expressed by follow equations:

(16) $$H_{\gamma }^{\mu }\left(\mathrm{GPa}\right)\;={\;\mathrm{AP}}^{\mu }{\left(\gamma_{b}^{\mu }\right)}^{-\frac{5}{3}},\;A\;=\;740$$

(17) $$\gamma_{b}^{\mu }\;=\;{\left(d^{\mu }\right)}^{3}/\underset{\mu }{\sum }{\left(d^{\mu }\right)}^{3}N_{b}^{\mu },{\;N}_{b}^{\mu }\;={\;N}^{\mu }/\Omega$$

where $P^{\mu }$ is the overlap population of μ-type bond; $H_{\gamma }^{\mu }$ is the hardness of μ-type bond; ${\;N}_{b}^{\mu }$ is the bond density per cubic angstroms of μ-type bond; d^μ^ is the bond length of μ-type bond; Ω is the cell volume. The hardness of the metallic bond is not considered in this model, because the calculation results of metallic bonds cannot be calculated accurately in the first-principle calculations. The electrons of the metallic bonds are shared in the whole material. The hardness of metallic bonds is not taken into account in the hardness model. The Gao’s hardness of materials can be expressed by the geometric average of all bond hardness, which is shown as follows:

(18) $$H_{\gamma }^{\mu }\left(\mathrm{GPa}\right)\;=\;{\left[{\left(H_{\gamma }^{\mu_{1}}\right)}^{m_{1}}{\left(H_{\gamma }^{\mu_{2}}\right)}^{m_{2}}\ldots {\left(H_{\gamma }^{\mu_{n}}\right)}^{m_{n}}\right]}^{\frac{1}{m_{1}+m_{2}+m_{3}}}.$$

where m_n_ is the bond number of μ-type bond.

**Figure 3:**
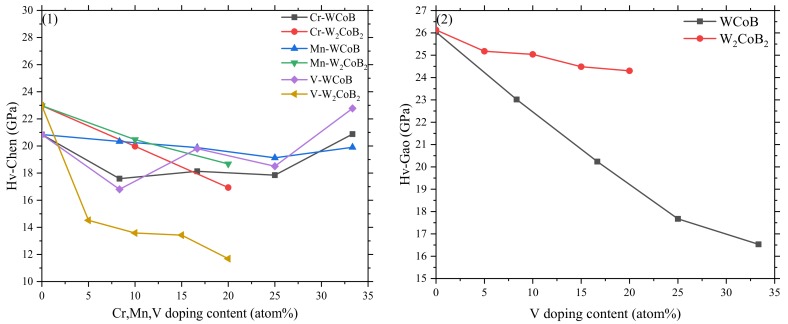
Plots of theoretical hardness versus V doping content of W_4_Co_4−*x*_(Cr, Mn, V)*_x_*B_4_ and W_8_Co_4−*x*_V*_x_*B_8_ (*x* = 0, 1, 2, 3, 4), obtained by different models: (**1**) Hv-Chen; (**2**) Hv-Gao.

The vickers hardness of V doped WCoB and W_2_CoB_2_ is shown in Figure 3, which are named Hv-Chen and Hv-Gao. The hardness models are usually based on the binary oxide, so the hardness is characterized by different models to explore the variation of the hardness of V doped WCoB and W_2_CoB_2_ ternary boride.

For WCoB ternary boride, the hardness varies around the hardness of the undoped structure. However, the mechanical properties have been studied in the previous section, and the bulk modulus decreases with increasing V doping content. It is obvious that the variation trend of Hv-Chen is similar to the variation trend of the shear modulus, which indicates that the shear modulus plays an important role in the hardness. The symmetrical doped structure has a relatively higher hardness than the unsymmetrical structure, implying better performance of symmetrical structures. However, Gao’s model shows that V doping leads to a decreasing in hardness, which is consistent with population analysis.

For W_2_CoB_2_ ternary boride, it is obvious that all hardness models show that V doping contributes to the decrement of hardness. It is more likely to be caused by the larger atom radius of V (r = 171 pm) than by Co (r = 152 pm), which leads to the weaker B–V covalent bonds and W–V metallic bonds. The Gao’ model especially shows that decreasing amplitude is little in W_2_CoB_2_ ternary boride.

Compared with the effect of Cr and Mn doped WCoB and W_2_CoB_2_ [35,36], we can assume Cr doped WCoB or V doped W_2_CoB_2_ are the valuable choices of the experiment because Gao’s model depends on the type of cell, and the previous studies are based on the unit cell of W_2_CoB_2_. So only Hv-Gao of V doped WCoB and W_2_CoB_2_ is shown in Figure 3. The mechanical properties and Vickers hardness of Cr, Mn, V doped WCoB and W_2_CoB_2_ indicates Cr doped WCoB and Mn doped W_2_CoB_2_ are the best choice of doping element and ternary boride, which have high ductility, hardness and relatively high mechanical properties.

### 3.4. Density of States

**Figure 4:**
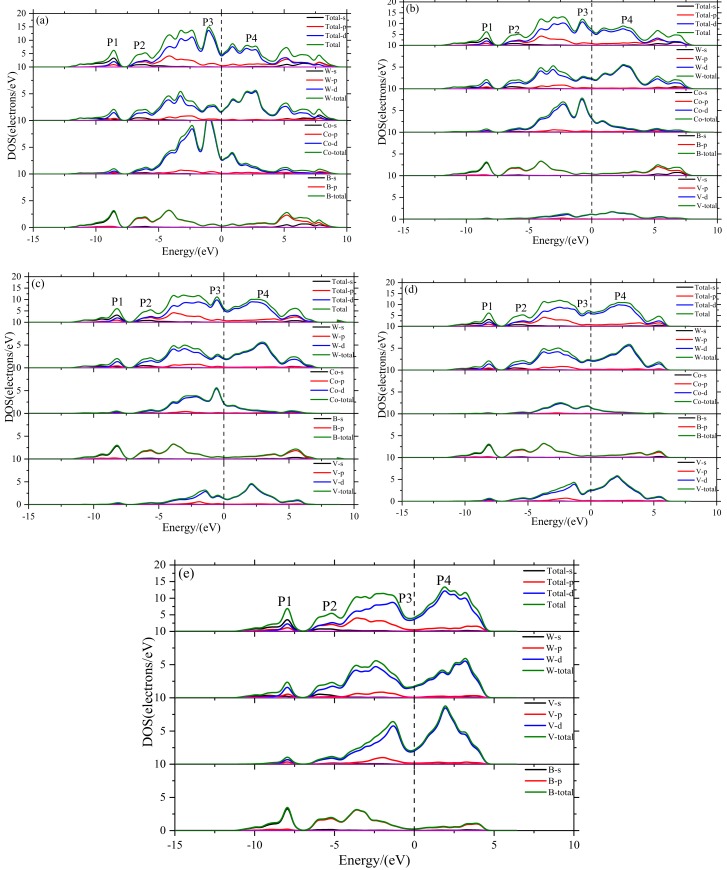
Plots of total and partial density of states of WCoB with different Mn doping content ((**a**) W_4_Co_4_B_4_; (**b**) W_4_Co_3_VB_4_; (**c**) W_4_Co_2_V_2_B_4_; (**d**) W_4_CoV_3_B_4_; (**e**) W_4_V_4_B_4_).

**Figure 5:**
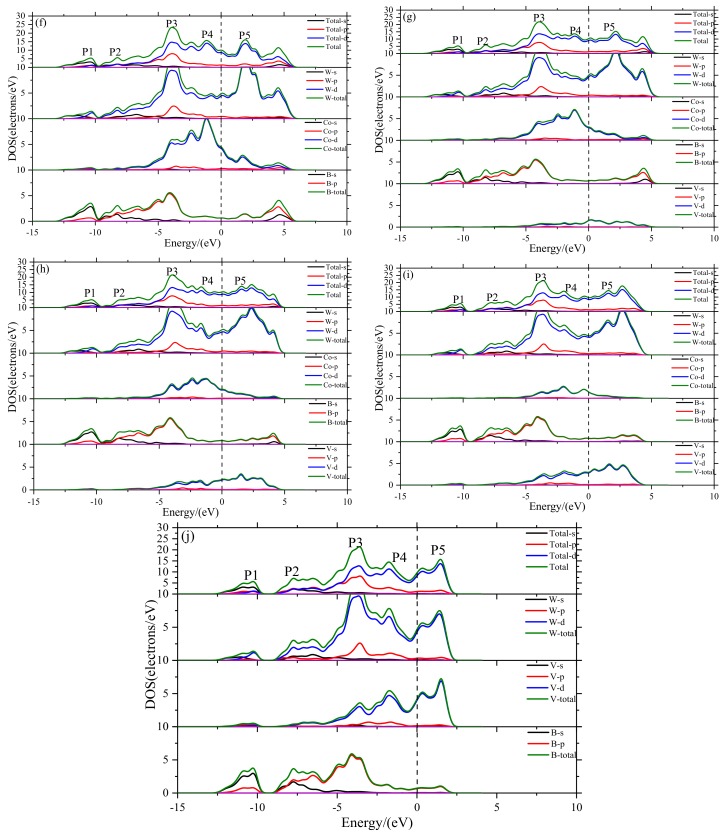
Plots of total and partial density of states of WCoB with different V doping content ((**f**) W_8_Co_4_B_8_; (**g**) W_8_Co_3_VB_8_; (**h**) W_8_Co_2_V_2_B_8_; (**i**) W_8_CoV_3_B_8_; (**j**) W_8_V_4_B_8_).

The nature of mechanical properties can be revealed by studying the electronic structure and chemical bonding features. The total density of states (DOS) and partial density of states (PDOS) of V doped WCoB and W_2_CoB_2_ are illustrated in Figure 4 and Figure 5. The black vertical dashed line represents the Fermi level and the deep valley around the Fermi level is named the pseudogap (E_p_). The orbitals above the pseudogap are usually composed of d-d orbitals and contribute to the formation of metallic bonds. The larger displacement between the pseudogap is related to the formation of covalent bonds and the weakness of metallic bonds. Yu [53] found a higher value of density of states at Fermi level indicating the increasing stability of non-crystal and quasi-crystal. However, the stability of typical crystals, including WCoB and W_2_CoB_2_, cannot be judged by the value of density of states at Fermi level.

The calculated DOS and PDOS of V doped WCoB are illustrated in Figure 4. In a range from −15 eV to 10 eV, there are several peaks which can be divided into different peaks. So, we chose some typical peaks to discuss the effect of V doping on WCoB. We named P1, P2, P3, P4 and P5 for peaks located at −8.6 eV, −6.0 eV, −1.0 eV, 0.83 eV, 1.9 eV. Because P1 is composed of B-2s orbital and W-5d orbital and P2 is composed of B-2p orbital and W-5d orbital, it is obvious that P1 and P2 varies little with increasing V doping content. By viewing Figure 4a, we can see that the location of B-2s orbital and B-2p orbital are away from Fermi level. These peaks change little with increasing V doping content and mainly form a covalent bond between B–W, B–Co and B–V, which gives a positive contribution to the shear modulus and hardness by enlarging the pseudogap. Because there exists a strong hybridization between B-2p orbital, V-3d orbital, Co-3d orbital and W-5d orbital, some peaks vanish with increasing V doping content.

However, P3, P4 and P5, which are close to the Fermi level, are obviously affected by the V doping content. One of the most obvious features of compounds is the metallic character at Fermi level, and V doping leads to a significant change at Fermi level and the formation of metal-to-metal bonding between V, Co and W. P3 is composed by Co-3d orbital and W-5d orbital, and the decrement of Co content leads to the decreasing of P3. Because P4 is composed of Co-3d orbital, W-5d orbital and V-3d orbital, the variation of P4 is minor. However, the influence of Co-3d orbital is greater than V-3d orbital, so the decrement of Co content contributes to the vanishing of P4. P5 is composed of V-3d orbital and W-5d orbital, so increasing the V doping content leads to increasing P5.

By comparing Figure 4 and Figure 5, it is found that DOS and PDOS of V doping W_2_CoB_2_ are similar to those of V doping WCoB. We can divide the DOS into several typical peaks, which can be named P1, P2, P3, P4 and P5, located at −10.4, −7.7, −3.9, −1.2 and 1.9 eV. It is clear that P1 is made up of the strongly hybridized B-2s and W-5d orbitals. Similarly, P2 is composed by B-2p orbital and W-5d orbital. So, the values of P1 and P2 change little with increasing V doping content. P1 and P2 exist a resonance peak between B-2s and B-2p orbitals, which means the formation of the B–B covalent bond and contributes to the shear modulus and hardness.

P3 is mainly composed of W-5d orbit with a small amount of Co-3d, V-3d and B-2p orbitals. Because increasing V content compensates the decreasing Co content, so the intensity of P3 varies little in different structures. We notice that the intensity of P4 and P5 obviously vary in different structures. P4 is mainly affected by Co-3d orbital and P5 is mainly affected by V-3d orbital, so the increasing V doping content leads to a decrease of P4 and an increase of P5. P3, P4 and P5 mainly form metallic bonds, which have a negative influence on the shear modulus.

Based on the DOS and PDOS of WCoB and W_2_CoB_2_, it is clear that V doping leads to strong hybridization between B and V orbitals, which leads to an increase of overlap population. The formation of weaker B–V covalent bonds and W–V metallic bonds contributes to a decrease of the shear modulus and hardness. The analysis of the electronic structure is consistent with hardness. If V doping cannot lead to a significantly solid solution or grain refinement [27,28], V doping may not be a suitable choice of transition doping element.

### 3.5. Charge Density Difference

**Figure 6:**
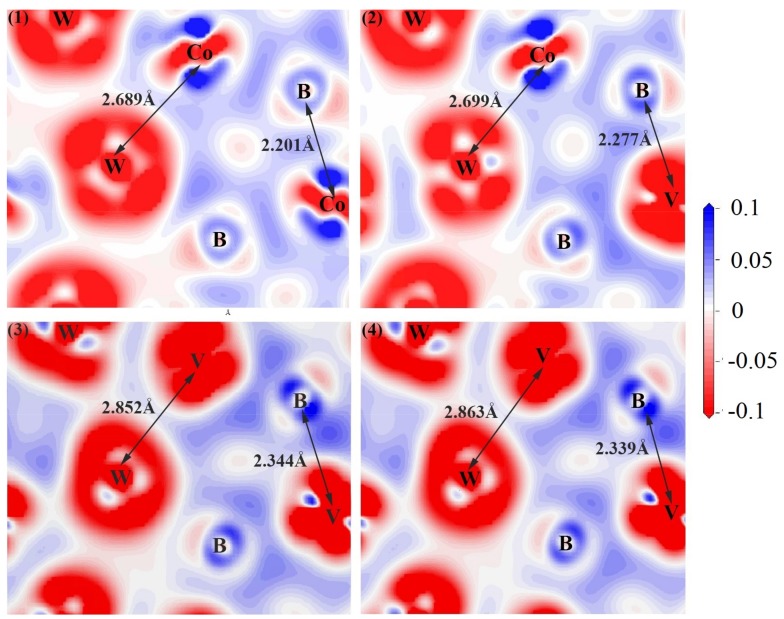
Illustration of charge density difference of V doped WCoB at different structure: (**1**) W_4_Co_4_B_4_-a; (**2**) W_4_Co_3_VB_4_-b; (**3**) W_4_CoV_3_B_4_-d; (**4**) W_4_V_4_B_4_-e.

**Figure 7:**
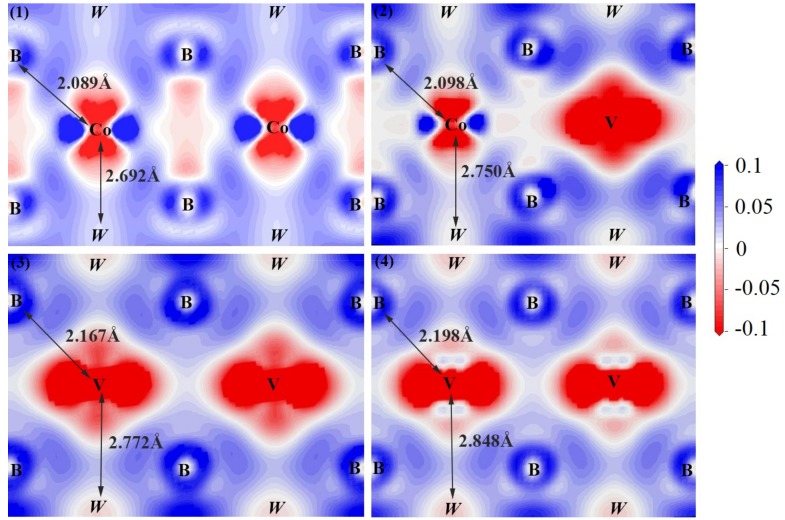
Illustration of charge density difference of V doped W_2_CoB_2_ at different structure: (**1**) W_8_Co_4_B_8_-f; (**2**) W_8_Co_3_VB_8_-g; (**3**) W_8_Co_2_V_2_B_8_-h; (**4**) W_8_V_4_B_8_-j.

To further explore the bond characterization, the charge density difference of V doped WCoB and W_2_CoB_2_ was calculated by first-principle calculation, which is shown in Figure 6 and Figure 7. The charge density differences of V doped WCoB and W_2_CoB_2_ are shown along the (010) and (100) plane for different doping contents, respectively. The crucial bonds and atoms are labeled in the figure and the italic atoms are not on the cross-section, which are the projection on the cross-section. Because all the crystal structures have a periodic arrangement, the charge density difference can be visualized by different cross-sections. For the charge density distribution, the red color implies the maximum localization of electrons; the white color means the electron density is almost zero; and the blue color indicates the maximum delocalization of electrons [54].

It is obvious that B–Co/V covalent bonds and W–Co/V metallic bonds are typical bonds in V doped WCoB ternary boride. With increasing V doping content, the bond length of B–Co/V covalent bonds increases from 2.201 Å to 2.339 Å. This phenomenon is caused by the larger atom radius of V atoms, resulting in increases of cell volume. When the doping content reaches up to 33.3 atom%, V atoms will have completely replaced Co atoms, resulting in the increment of symmetry and a slight decrement of bond length. The bond length of W–Co/V metallic bonds obviously increase in the higher V doping content structure, and the weaker W–V metallic bonds lead to the decrement of shear modulus and hardness, which is consistent with the analysis of mechanical properties.

For V doped W_2_CoB_2_ ternary boride, the charge density difference is shown in Figure 7, which is mainly formed by B–Co/V covalent bonds and W–Co/V metallic bonds. With increasing V doping content, the bond lengths of B–Co/V and W–Co/V are up to 2.198 Å and 2.848 Å, which indicates that V doping is harmful to the bond hardness. The formation of weaker bonds leads to the decrease of shear modulus and hardness, which is consistent with the analysis of DOS. When we consider Figure 3 and Figure 4, the V doping contributes to the formation of W–W metallic bonds, indicating the increase of orbital hybridization, which is also consistent with average overlap population analysis.

The calculated charge density difference and electronic structures show that V doping leads to the formation of weaker B–V covalent bonds and W–V metallic bonds with the adjacent atoms. The decrease of the shear modulus and hardness are attributed to increasing bond length. Although population increases slightly in the V doped structure, it cannot offset the decrease of the number of stronger B–Co covalent bonds and W–Co metallic bonds. Therefore, V doping leads to the decrease of mechanical properties and hardness.

## 4. Conclusions

In summary, comparing with Cr, Mn doped WCoB and W_2_CoB_2_, the stability, lattice constants, mechanical properties, average overlap population, hardness, density of states and the charge density difference of V doped WCoB and W_2_CoB_2_ ternary boride are calculated and discussed based on the first-principle calculation, which can be drawn as follows.

(1) By analyzing the cohesive energy, formation energy, we can see that all V doped structures are stable. With increasing V doping content, stability increases slightly when the V doping content is up to 16.67 at.% for WCoB and 20 at.% for W_2_CoB_2_. However, the formation energy shows V doped WCoB and W_2_CoB_2_ ternary boride are harder to form except for the W_4_V_4_B_4_ structure. The larger atom radius of V atoms leads to the increase of lattice constants.
(2) The mechanical properties, average overlap population and hardness show V doping leads to the decreasing of shear modulus and hardness, which is attributed to the weaker B–V covalent bonds and W–V metallic bonds. Two different hardness models show the shear modulus is closely related to the hardness, and V doped W_2_CoB_2_ has higher ductility.
(3) The electronic structure of V doped WCoB and W_2_CoB_2_ ternary boride is studied by DOS, PDOS and charge density difference. The formation of weaker B–V covalent bonds and W–V metallic bonds contributes to the orbital hybridization, which is inconsistent with the analysis of mechanical properties and hardness. Moreover, the formation of W–W metallic bonds at high V doping content is harmful to the shear modulus and mechanical properties.

## Figures and Tables

**Table 1:** Calculated results of lattice parameters, cohesive energy E_coh_ (eV/atom), impurity formation energy E_f_ (eV/atom), and formation enthalpy ΔH of W_4_Co_4−*x*_V*_x_*B_4_ and W_8_Co_4−*x*_V*_x_*B_8._

WCoB/W_2_CoB_2_	Lattice Constants (Å)	V (Å^3^)	E_coh_	E_f_	∆H
W_4_Co_4_B_4_-ex	a = 5.746, b = 3.203, c = 6.652	122.43	-	-	-
a-W_4_Co_4_B_4_	a = 5.745, b = 3.256, c = 6.624	123.880	−9.311	0	−0.494
B–W_4_Co_3_VB_4_	a = 5.830, b = 3.210, c = 6.798	127.213	−9.299	0.149	−0.460
c-W_4_Co_2_V_2_B_4_-sy	a = 5.973, b = 3.162, c = 7.010	130.178	−9.342	−0.370	−0.481
c-W_4_Co_2_V_2_B_4_-unsy	a = 5.919, b = 3.162, c = 6.974	130.437	−9.321	−0.122	−0.460
d-W_4_CoV_3_B_4_	a = 5.937, b = 3.137, c = 7.165	133.475	−9.367	−0.673	−0.484
e-W_4_V_4_B_4_	a = 5.953, b = 3.113, c = 7.345	136.118	−9.448	−1.644	−0.543
W_4_Co_2_B_4_-ex	a = 7.075, b = 4.564, c = 3.177	102.59	-	-	-
f-W_8_Co_4_B_8_	a = 7.106, b = 4.565, c = 3.189	103.454	−9.486	0	−0.531
g-W_8_Co_3_VB_8_	a = 7.182, b = 4.643, c = 3.156	105.260	−9.462	0.467	−0.492
h-W_8_Co_2_V_2_B_8_	a = 7.175, b = 4.670, c = 3.166	106.750	−9.469	0.332	−0.483
i-W_8_CoV_3_B_8_	a = 7.212, b = 4.794, c = 3.140	108.566	−9.472	0.274	−0.470
j-W_8_V_4_B_8_	a = 7.189, b = 4.860, c = 3.151	110.094	−9.505	−0.386	−0.487

**Table 2:** The calculated elastic constants (in GPa) of W_4_Co_4−*x*_V*_x_*B_4._

	a-W_4_Co_4_B_4_	B–W_4_Co_3_VB_4_	c-W_4_Co_2_V_2_B_4_	d-W_4_CoV_3_B_4_	e-W_4_V_4_B_4_
C_11_	551.84	493.02	524.73	515.56	556.79
C_22_	515.90	475.98	484.01	487.05	497.32
C_33_	609.34	558.45	549.28	514.86	522.41
C_44_	234.37	216.15	219.46	210.78	220.10
C_55_	190.14	169.89	165.62	141.02	149.42
C_66_	236.81	221.97	237.74	212.29	228.58
C_12_	264.24	270.44	242.05	229.14	206.52
C_13_	177.11	180.14	161.29	154.40	127.24
C_23_	225.26	220.23	206.40	190.04	177.06

**Table 3:** The calculated elastic constants (in GPa) of W_8_Co_4−*x*_V*_x_*B_8._

	a-W_4_Co_4_B_4_	B–W_4_Co_3_VB_4_	c-W_4_Co_2_V_2_B_4_	d-W_4_CoV_3_B_4_	e-W_4_V_4_B_4_
C_11_	589.86	544.95	538.71	517.45	545.80
C_22_	608.81	561.13	564.37	565.08	586.25
C_33_	542.51	490.30	482.64	482.50	499.95
C_44_	241.93	241.06	239.58	244.70	257.87
C_55_	245.71	228.43	227.96	244.34	235.65
C_66_	210.82	95.46	76.37	63.87	37.31
C_12_	162.49	158.43	156.86	156.72	133.52
C_13_	250.69	261.84	258.16	258.26	238.41
C_23_	238.90	248.74	232.46	212.01	195.72

**Table 4:** Calculated overlap population of bonds for W_4_Co_4−*x*_V*_x_*B_4_.

	a-W_4_Co_4_B_4_	B–W_4_Co_3_VB_4_	c-W_4_Co_2_V_2_B_4_	d-W_4_CoV_3_B_4_	e-W_4_V_4_B_4_
B–B	-	-	-	-	-
B–Co	0.307	0.309	0.303	0.300	-
B–W	0.530	0.558	0.565	0.592	0.603
B–V	-	0.123	0.157	0.156	0.157
Co–Co	−0.150	−0.140	−0.0900	-	-
Co–W	−0.055	−0.0100	−0.0150	0.0575	-
Co–V	-	−0.330	-	−0.220	-
W–W	−0.055	−0.055	0.0300	0.100	0.150
W–V	-	−0.240	−0.1125	−0.0950	−0.0425
V–V	-	-	−0.530	−0.410	−0.33

**Table 5:** Calculated overlap population of bonds for W_8_Co_4−*x*_V_*x*_B_8_.

	a-W_8_Co_4_B_8_	B–W_8_Co_3_VB_8_	c-W_8_Co_2_V_2_B_8_	d-W_8_CoV_3_B_8_	e-W_8_V_4_B_8_
B–B	0.590	0.5925	0.575	0.568	0.580
B–Co	0.210	0.237	0.27	0.29	-
B–W	0.200	0.201	0.208	0.208	0.207
B–V	-	0.13	0.14	0.187	0.220
Co–Co	-	-	-	-	-
Co–W	0.0833	0.0933	0.113	0.127	-
Co–V	-	-	-	-	-
W–W	−0.0833	−0.0492	0	0.0445	0.0267
W–V	-	−0.01	0.0167	0.04	0.0867
V–V	-	-	-	-	-
